# Nitrogen-Vacancy Centers in Fluorescent Nanodiamonds: Emerging Applications from Healthcare Diagnostics to Semiconductor Metrology

**DOI:** 10.1021/acs.accounts.6c00047

**Published:** 2026-06-04

**Authors:** Huan-Cheng Chang

**Affiliations:** † Institute of Atomic and Molecular Sciences, 38017 Academia Sinica, Taipei 106, Taiwan; ‡ Department of Chemical Engineering, National Taiwan University of Science and Technology, Taipei 106, Taiwan; § Department of Chemistry, National Taiwan Normal University, Taipei 106, Taiwan

## Abstract

Nitrogen-vacancy (NV) centers in diamond are a unique class of quantum defects distinguished by their exceptional optical and magnetic properties, including bright photoluminescence, outstanding photostability, and optically addressable spin states. In nanoscale diamonds, commonly referred to as fluorescent nanodiamonds (FNDs), NV centers can exist in both neutral (NV^0^) and negatively charged (NV^–^) states. These nanoparticles are chemically inert, highly biocompatible, and readily amenable to surface functionalization. Together, these attributes have established FNDs as powerful biological quantum probes since their introduction in 2005. Notably, the same optical and spin properties that enable their success in biological environments also underpin their utility in more demanding physical and engineering contexts. This Account highlights three emerging applications that arise from this shared foundation: immunodiagnostics, extreme ultraviolet (EUV) metrology, and semiconductor device analysis.

First, we show that FNDs with NV^–^ centers are excellent fluorescent quantum reporters for quantitative immunoassays employing antibodies for specific antigen detection. A key limitation of traditional fluorescence-based immunoassays is the high background from substrates like nitrocellulose membranes, which significantly reduces detection sensitivity. This challenge is overcome by combining laser excitation with lock-in detection of magnetically modulated fluorescence from NV^–^ centers in FNDs to effectively eliminate background interference. The approach has enabled highly sensitive, high-throughput, quantitative, and rapid biomarker detection, marking a practically useful application of NV quantum defects in healthcare diagnostics.

Second, FNDs with NV^0^ centers have emerged as a novel type of scintillator for EUV sensing and imaging. These carbon-based scintillators are fabricated into uniform, thin, and chemically stable films using electrospray deposition. They emit bright red fluorescence from NV^0^ centers when FNDs are exposed to EUV radiation at wavelengths of 13.5 nm. The scintillators are nonhygroscopic, photostable, and compatible with fiber-optic plates and sensors, allowing for integration into compact, high-resolution detection systems. These properties render them highly suitable for real-time, long-term imaging in the EUV and soft X-ray regimes, particularly for photolithographic applications.

Third, FNDs with NV^–^ centers are quantum sensors capable of measuring temperature, magnetic, and electric fields at the nanoscale. These measurements are crucial for the design and evaluation of next-generation semiconductor devices. An innovative technique, termed FND-based lock-in photoluminescence thermography, has been developed to enable wide-field, real-time temperature mapping of actively operating devices such as bipolar junction transistors and field-effect transistors. The method achieves nanometer-scale spatial resolution and millisecond temporal resolution, yielding valuable insights into heat generation and dissipation processes in operando semiconductor devices.

In summary, NV centers in FNDs constitute robust platforms for quantum sensing and metrology across a broad range of domains. From enhancing the sensitivity of immunodiagnostics to advancing EUV imaging and improving semiconductor thermal analysis, these quantum defects serve as transformative tools at the intersection of materials chemistry, biomedicine, and quantum technologies.

## Graphical abstract

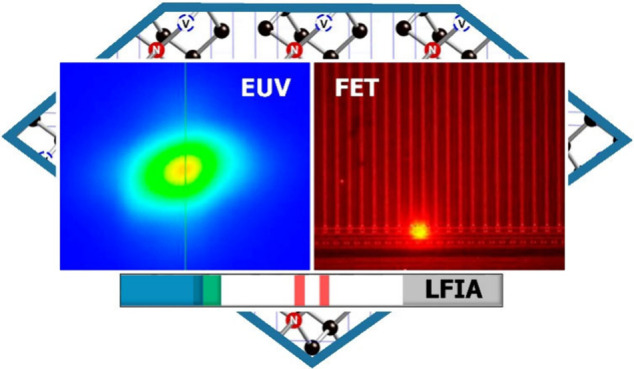

## Key References

-

Hui, Y. Y. ; Chen, O. J. ; Lin, H.-H. ; Su, Y.-K. ; Chen, K. Y. ; Wang, C.-Y. ; Hsiao, W. W.-W. ; Chang, H.-C. Magnetically Modulated Fluorescence of Nitrogen-Vacancy Centers in Nanodiamonds for Ultrasensitive Biomedical Analysis. Anal. Chem. 2021, 93, 7140–7147 33913330 10.1021/acs.analchem.1c01224.^1^ This work develops a magnetic modulation method for FNDs with NV^–^ centers as reporters in immunoassays, enabling highly selective and sensitive detection of disease markers for healthcare diagnostics.
-

Yang, T.-I. ; Hui, Y.-Y. ; Lo, J.-I. ; Huang, Y.-W. ; Lee, Y.-Y. ; Cheng, B.-M. ; Chang, H.-C. Imaging Extreme Ultraviolet Radiation Using Nanodiamonds with Nitrogen-Vacancy Centers. Nano Lett. 2023, 23, 9811–9816 37708490 10.1021/acs.nanolett.3c02472.^2^ This paper reports the pioneering development and application of uniform thin films of FNDs with NV^0^ centers for sensing and imaging EUV radiation, providing a tool essential for beam diagnostics in advanced semiconductor manufacturing.
-

Hui, Y. Y. ; Tsui, Y.-M. ; Tang, Y.-X. ; Chang, H.-C. Ultrathin Fluorescent Nanodiamond Films for Nanoscale Quantum Sensing in Operando Semiconductor Devices. Adv. Funct. Mater. 2026, 36, e13406 .^3^ This study applies ultrathin FND films containing both NV^0^ and NV^–^ centers to the surfaces of semiconductor devices for thermal sensing and management, addressing critical challenges in electronics miniaturization.

## 1 Introduction

Diamonds with nitrogen-vacancy (NV) centers are among the most practical quantum-enabled materials available to date, offering wide-ranging applications in quantum sensing, nanoscale imaging, and foundational studies in the physical sciences. Research on these centers dates back to the 1960s, when scientists first observed the absorption and emission spectra of diamonds following electron irradiation and thermal annealing.^4^ These initial studies were motivated by the desire to understand the origins of diamond coloration, a topic of central interest in both gemology and materials science.^5^ For single-crystal diamonds synthesized using high-pressure high-temperature (HPHT) methods, the treatments can induce a color change from yellow to red. Later investigations revealed that the diamond coloration process involves lattice damage and the generation of structural defects, such as carbon vacancies,^6^ which form stable complexes with impurities, including nitrogen atoms, in the substrate. The identification of these color centers as NV defects in the 1970s marked a significant milestone in the field.^4^ Since then, NV centers have been extensively investigated both theoretically and experimentally to uncover their intrinsic optical and magnetic properties.^7^ Over the last two decades, research efforts have progressed from fundamental spectroscopic studies to sophisticated applications of NV centers across physics, biology, materials science, and, more recently, advanced quantum technologies.^8–11^

The NV center in diamond is a point defect consisting of a substitutional nitrogen atom next to a carbon vacancy within the crystal lattice. It can exist in different charge states, with NV^0^ (neutral) and NV^–^ (negatively charged) being the most common. Both of these structural defects possess unique electronic states within the diamond’s band gap, which spans 5.47 eV in energy (227 nm in wavelength). They each display characteristic zero-phonon lines (ZPLs) in their absorption and fluorescence spectra with ZPL = 2.156 eV (575 nm wavelength) for NV^0^ and ZPL = 1.945 eV (637 nm wavelength) for NV^–^.^12,13^ Owing to their remarkable physicochemical stability, NV centers have been successfully implanted and detected in synthetic diamonds produced by HPHT and chemical vapor deposition (CVD) methods, as well as in nanoscale diamonds. Their widespread availability has facilitated diverse applications of these atom-like color centers across multiple fields of science, technology, and engineering.

Fluorescent nanodiamonds (FNDs) are monocrystalline diamond particles containing NV centers at concentrations of up to 10 ppm. They can be produced in high yield through ion or electron irradiation, followed by thermal annealing under a vacuum.^14,15^ Originally developed in 2005 for bioimaging applications,^16^ FNDs have since been widely recognized for their high fluorescence brightness, low cytotoxicity, and ease of surface modification for biomolecule conjugation. These features, along with excellent photostability and remarkable quantum properties of NV centers, make FNDs ideal for biological applications, including long-term cell tracking, super-resolution imaging, nanoscale temperature sensing,^17,18^ and magnetic relaxometry,^19^ all of which have been extensively explored over the past two decades. Recently, new research opportunities have emerged across three diverse and exciting areas: immunodiagnostics, extreme ultraviolet (EUV) metrology, and semiconductor device analysis. Specifically, FNDs enable ultrasensitive biomolecular detection in immunodiagnostics, facilitate nanoscale quantum sensing of thermal and magnetic fields in semiconductor device analysis, and can withstand high-energy radiation, ensuring reliable measurements in EUV metrology. Collectively, these capabilities establish FNDs as a unique quantum platform bridging biological, photonic, and electronic applications. This Account highlights the potential of FNDs and the major developments across these fields.

## 2 NV^0^ and NV^–^ Centers

Figure 1a presents an electronic energy diagram for N-doped diamond.^20^ Both NV^0^ and NV^–^ centers possess isolated electron spins within the diamond lattice. Specifically, these centers are characterized by one and two unpaired spins in their ground states, labeled as ^2^ *E* for NV^0^ and ^3^ *A*_2_ for NV^–^. Compared to the energy level of N^0^, which is located 1.7 eV below the conduction band minimum, the ground electronic state of NV^–^ lies 0.9 eV lower. The exact energy level of NV^0^ has yet to be experimentally determined, but theoretical predictions place it ∼1.2 eV above the valence band maximum.^21^ When excited by a green laser, both NV^0^ and NV^–^ centers emit bright red fluorescence, but they can be easily distinguished by their characteristic ZPLs, as shown in Figure 1b.

**Figure 1:**
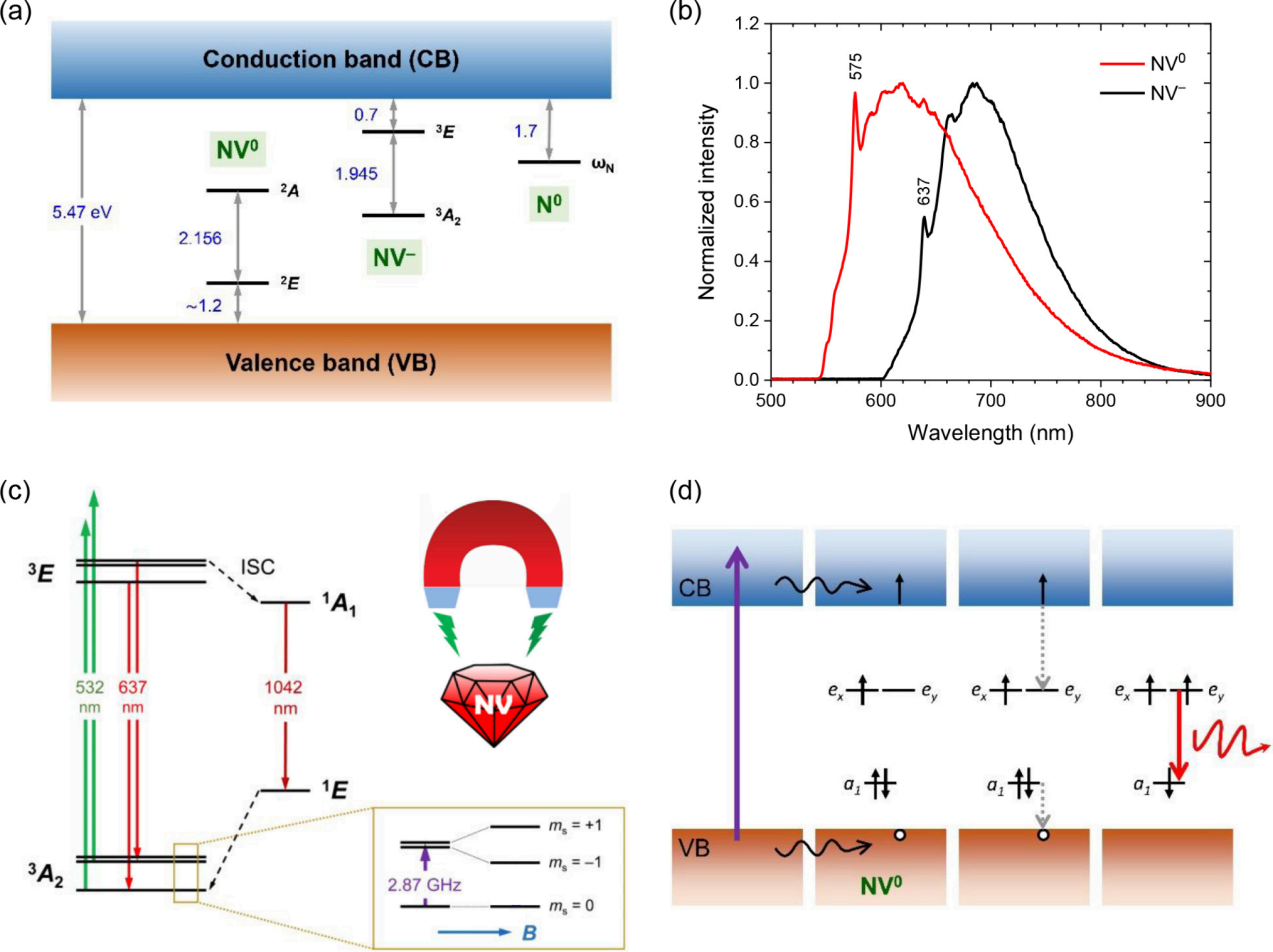
(a) Relative energy levels of N^0^, NV^0^, and NV^–^ defects in the band gap of a diamond. All numbers are in units of eV. (b) Normalized fluorescence spectra of NV^0^ and NV^–^ centers in diamonds with their characteristic ZPLs at 575 and 637 nm, respectively. (c) Energy level diagram of NV^–^ in diamond and its energy relaxation pathways upon photoexcitation at 532 nm in the presence of an external magnetic field (*B*). ISC: intersystem crossing. (d) Above-band-gap excitation of diamond and subsequent electron–hole recombination through an NV^0^ center.

An important characteristic of the NV^–^ center is that its spin states can be optically polarized and coherently manipulated using microwave fields. The electronic ground state (^3^ *A*_2_) of the center is a triplet, exhibiting a magnetic resonance frequency of 2.87 GHz for the Δ*m*_s_ = 0 → ±1 transitions (Figure 1c). When excited by a laser with a wavelength shorter than 637 nm, the center emits red fluorescence. At the same time, nonradiative relaxation also occurs via coupling of the *m*_s_ = ±1 sublevels in the excited state (^3^ *E*) with the metastable state (^1^ *A*_1_). A decay to the *m*_s_ = 0 sublevel of ^3^ *A*_2_ follows this intersystem crossing process, resulting in spin polarization. A notable manifestation of this effect is that the fluorescence intensity from the ^3^ *E* → ^3^ *A*_2_ transition reaches a maximum after several excitation cycles. However, in the presence of an external magnetic field that is neither aligned with nor perpendicular to the N–V axis, the fluorescence intensity decreases due to the lifting of degeneracy between the *m*_s_ = ±1 sublevels and their mixing with the *m*_s_ = 0 sublevel in both the ground and excited states. For an ensemble of NV^–^ centers in bulk diamond, this can produce a change in fluorescence intensity of more than 10% at a magnetic field strength of 30 mT due to spin depolarization.^22^ A similar effect can be induced by applying a microwave field with a frequency resonating with the Δ*m*_s_ = 0 → ±1 transitions. Importantly, these unique behaviors are preserved in NV^–^ centers within nanoscale diamonds, strongly suggesting that modulation of the magnetic or microwave field, combined with lock-in detection, can enable selective detection of FNDs in high-background environments.^23,24^

Although less characterized than NV^–^, NV^0^ plays a more prominent role in energy relaxation processes following above-band-gap excitation of diamonds using high-energy electrons and UV photons. A previous study on the two-photon excitation of FNDs with a 344 nm femtosecond laser has demonstrated that the pulsed UV excitation produces fluorescence exclusively from NV^0^ centers.^25,26^ The results closely resemble those of cathodoluminescence studies on FNDs with 60 keV electrons^27,28^ and are consistent with theoretical calculations predicting that the NV^0^ energy level is situated closer to the valence band maximum than that of NV^–^, thereby enabling faster acceptor ionization during electron–hole pair recombination (Figure 1d). The ability to efficiently convert high-energy radiation into visible photons makes diamonds with NV^0^ centers useful as high-performance scintillators for real-time beam monitoring of EUV and soft X-ray.

## 3 Immunodiagnostics

Immunodiagnostics encompasses a variety of techniques relying on the interactions between antibodies and antigens. Produced by the immune system, antibodies bind specifically to antigens on pathogens. They are highly effective tools for detecting diseases, infections, and other medical conditions by identifying specific biomarkers in samples such as blood and urine. Recent advancements in the field have introduced fluorescent nanoparticles (e.g., plasmonic nanoparticles, quantum dots, upconversion nanoparticles, and FNDs) to enhance the detection sensitivity of immunoassays.^29^ Among these, FNDs with NV centers exhibit unique quantum sensing properties and have been successfully used as reporters in various immunoassays, including turbidimetric, lateral flow, and microfiltration assays.^1,30–37^ We summarize here the latest developments in the field, with a special focus on spin-enhanced lateral flow immunoassay (SELFIA). These developments signify the successful integration of quantum sensing techniques into immunodiagnostic platforms for real-world applications.

The FNDs used in immunoassays are prepared by ion or electron bombardment of monocrystalline HPHT diamond powders containing 100–200 ppm of N^0^ impurities.^17^ Intradefect excitation of the particles with a laser at 532 nm yields fluorescence emission with an intensity ratio of about 1:6 between NV^0^ and NV^–^ centers (Figure 2a).^26^ The predominance of NV^–^ centers in the spectrum suggests that FNDs can be selectively and sensitively detected in high-background environments using the magnetic modulation technique, as discussed in the previous section. Membrane-based immunoassays provide an excellent platform to demonstrate the principle. In these assays, the substrate employed typically comprises a nitrocellulose membrane, which generates strong background signals upon 532 nm excitation.^1^ Moreover, its spectral features significantly overlap with NV fluorescence, particularly near the 650 nm region (Figure 2a). Magnetically modulated fluorescence offers an effective method for distinguishing between these two types of signals. When an FND sample is positioned near an electromagnet, a clear modulation of fluorescence intensity is observed upon exposure to an alternating magnetic field with a strength of *B* = 40 mT (Figure 2b). In contrast, the modulation amplitude of the nitrocellulose membrane at the same position is nearly undetectable (<0.005%), over 1000 times lower than that of FNDs (Figures 2c and 2d). Notably, the measured magnetic modulation depth (>10%) is substantially greater than that achieved using microwave modulation (∼4%) for FNDs.^30^ Additionally, the gap between the sample and the surface of the electromagnet can be as much as 5 mm, making this immunoassay platform more practically useful for routine operations. Given *B* = 40 mT, a magnetic modulation frequency of *f* = 102.4 Hz, and a data acquisition time of 30 s, the detection limit for 100 nm FNDs on the nitrocellulose membrane is 0.04 ng/mm^2^, which corresponds to 2 × 10^4^ particles/mm^2^, assuming a spherical particle shape. No photobleaching of the FND signals has been observed throughout the measurement period.

**Figure 2:**
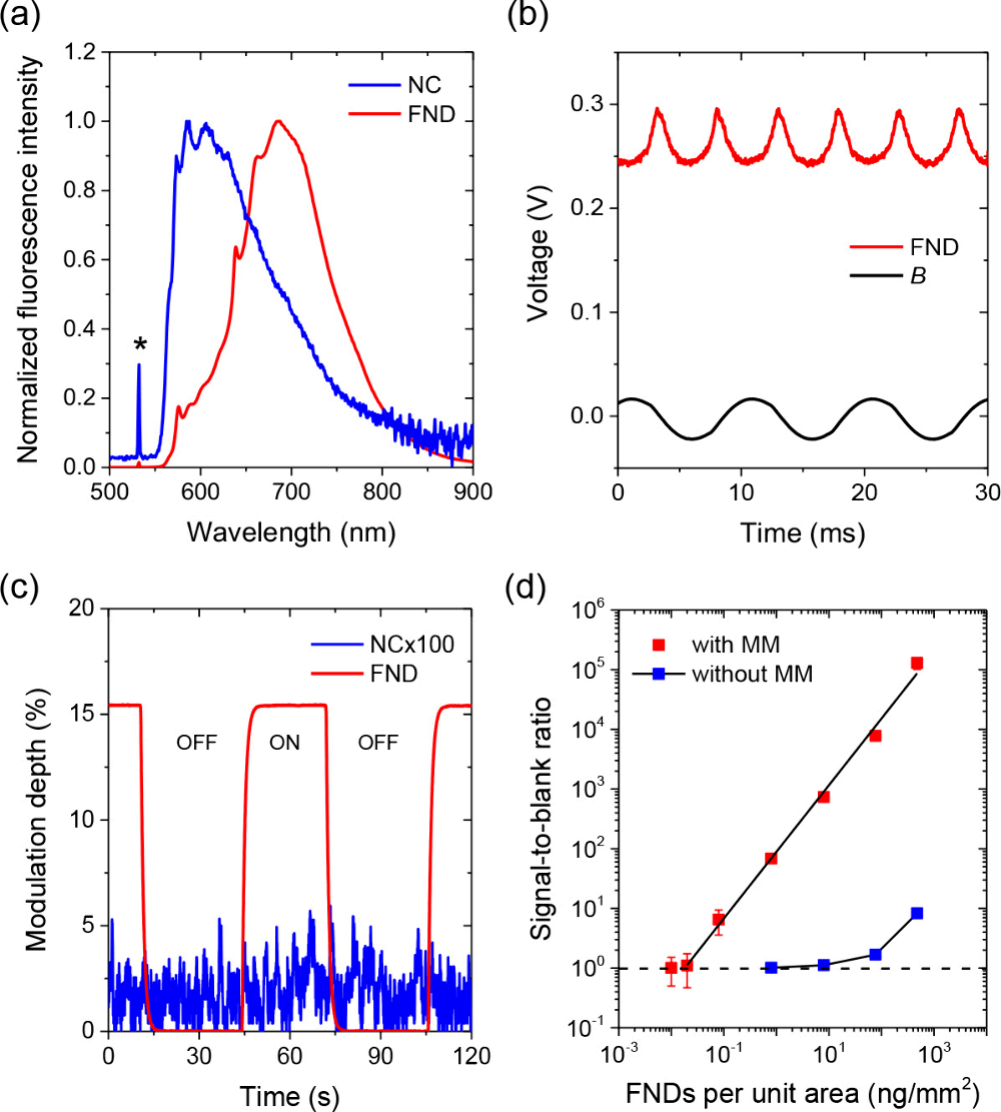
(a) Fluorescence spectra of FNDs and nitrocellulose (NC) membrane excited by a 532 nm laser. (b) Temporal profile of fluorescence signals from FNDs in response to an alternating magnetic field (*B*) measured using a Gaussmeter. (c) On/off modulation of the fluorescence intensities of FNDs deposited on an NC membrane strip exposed to an alternating magnetic field. (d) Detection of FNDs on an NC membrane strip with and without magnetic modulation (MM).

Figure 3a presents a schematic diagram of the SELFIA platform, which comprises an electromagnetic coil, a continuous-wave green laser, an objective lens, a long-pass filter, a photomultiplier tube, and a lock-in amplifier.^1^ To facilitate the practical use of FNDs as fluorescent reporters in immunoassays, the particles are first treated in molten salts to minimize size variability and surface roughness, thereby enhancing assay sensitivity and reproducibility.^33^ Transmission electron microscopy analysis of these FNDs, before and after KNO_3_ treatment at 500 °C for 1 h, clearly reveals the effects of oxidative etching on the diamond surface (Figure 3b).^33^ Dynamic light scattering measurements yield a number-averaged hydrodynamic diameter of 93 nm and a polydispersity index of 0.093 for the treated FNDs, which exhibit a more rounded shape. The diameter of these particles increases by about 10 nm following noncovalent conjugation with antigens or antibodies and subsequent blocking of unoccupied sites using bovine serum albumin (BSA). These nanoparticle bioconjugates are readily useful for immunoassays in direct, competitive, and sandwich formats (Figure 3c). An example of direct SELFIA is shown in Figure 3d for a well-characterized simple case, where biotinylated BSA is first conjugated to FNDs via noncovalent interactions and then captured by NeutrAvidin immobilized at the center of a nitrocellulose membrane strip via lateral flow. Magnetically modulated fluorescence measurements of the FND reporters successfully uncover an intense fluorescence band in the sample-scan profile. The approach has been extended to include antigen–antibody interactions for rapid antibody screening,^32^ an essential step in the immunodiagnostics of emerging infectious diseases.

**Figure 3:**
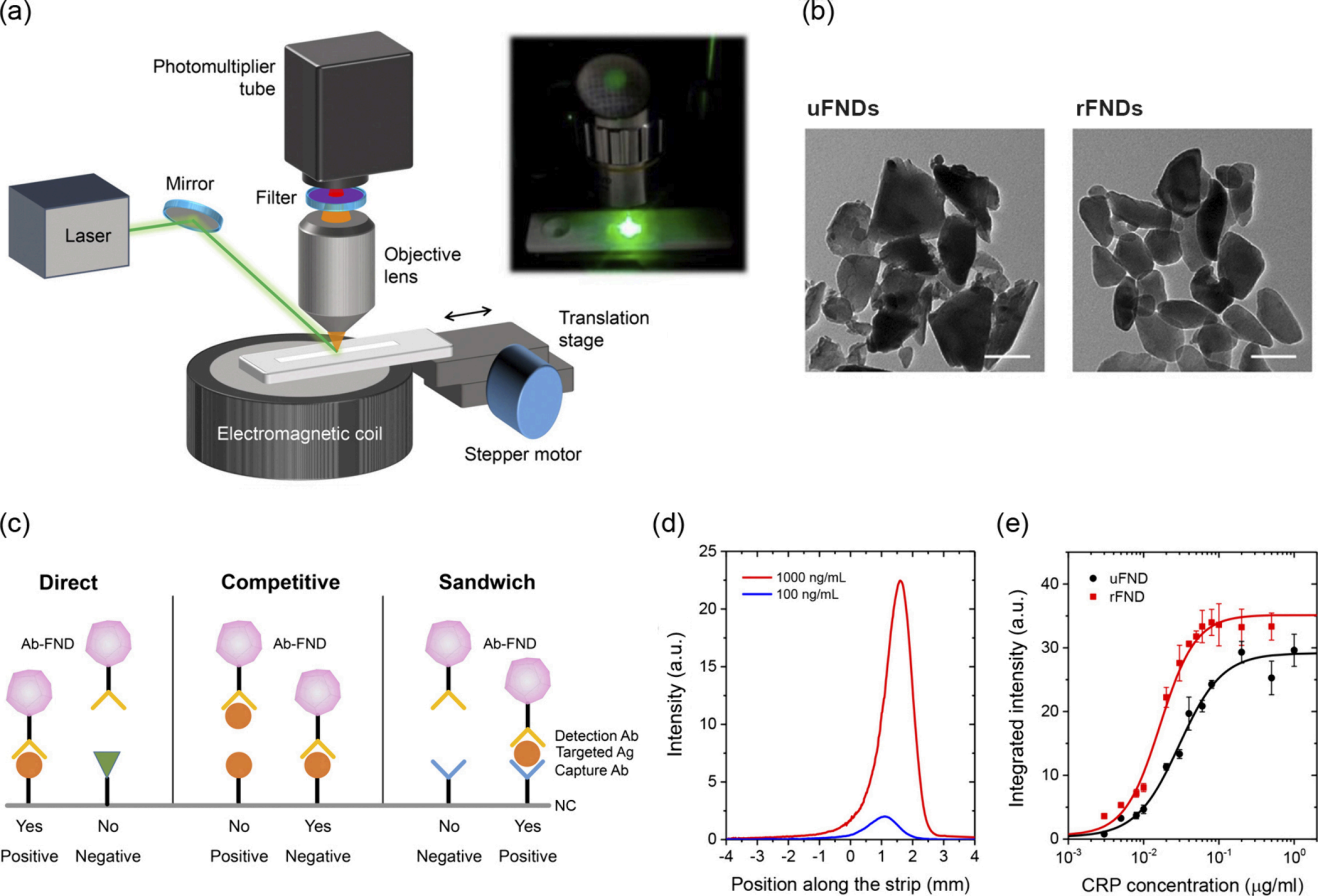
(a) Schematic diagram of the experimental setup for SELFIA. Inset: Photograph of SELFIA in operation near the objective lens region. (b) Transmission electron microscopy images of FNDs before (left) and after (right) a molten salt treatment. Scale bar: 200 nm. (c) Illustration of direct, competitive, and sandwich immunoassays using FNDs as reporters with negative and positive results. Ab: antibody, Ag: antigen, Ab-FND: antibody-conjugated FND, NC: nitrocellulose membrane. (d) Direct SELFIA of biotinylated BSA conjugated with FNDs and captured by NeutrAvidin on nitrocellulose membrane strips. The annotation indicates the two concentrations of FNDs fully coated with biotinylated BSA. (e) Sandwich SELFIA of CRP with pAb-P17 as both capture and detection antibodies. uFNDs: untreated FNDs, rFNDs: rounded FNDs.

The sandwich SELFIA platform has been successfully applied to the detection of diverse infectious agents and disease biomarkers, including C-reactive protein (CRP), SARS-CoV-2 spike proteins, dengue virus nonstructural proteins, and markers for tuberculosis and Alzheimer’s disease.^31–35^ Depending on the antibody pairs used in the assays, the typical detection limit for these antigens is 100 pg/mL when 100 μL of the sample solution is applied. This sensitivity is comparable to the ∼100 pg/mL detection limit specified by most commercial ELISA (Enzyme-Linked Immunosorbent Assay) kits for protein analytes.^38^ Figure 3e shows a representative result of the platform for detecting CRP, a liver-produced protein in response to inflammation. Its concentration rises rapidly during infection or other inflammatory events to 1–500 μg/mL, making it a valuable biomarker for assessing cardiovascular risk in clinical settings.^39^ Using the FND-based sandwich SELFIA, the detection of CRP at levels below 1 μg/mL can be easily achieved with only 3 μL of serum sample within a total assay time of 1 h. The SELFIA platform is rapid, sensitive, and readily adaptable for point-of-care testing.

## 4 EUV Metrology

EUV lithography is a cutting-edge photolithographic technique that uses EUV light to create extremely fine patterns on semiconductor wafers.^40,41^ Although the technique has become the standard for advanced semiconductor manufacturing, it remains costly and technically challenging. One of the key challenges is that EUV light is ionizing radiation with a shallow penetration depth in most materials.^42^ It often causes sample damage due to highly localized energy deposition, and consequently, reliable and durable diagnostic tools for this type of radiation have been lacking. YAG:Ce is a commonly used scintillator in X-ray computed tomography^43^ and has been applied to EUV imaging as well. However, its performance and applicability are limited by a long relaxation time, exceeding 100 ns.^44^ The discovery of diamonds with NV centers as scintillators for EUV sensing and imaging marks a significant advancement in the field.^2,45–48^ At 13.5 nm, which is the wavelength used in current EUV lithography, a CVD diamond with 0.3 ppm of NV centers exhibits a scintillation light yield of up to 7 photons/keV.^48^ Moreover, the scintillator shows a fluorescence lifetime of less than 30 ns.^26^ Although the penetration depth of the radiation into diamond is only about 0.1 μm,^49^ the material exhibits excellent chemical stability and photostability, making it highly attractive for EUV beam diagnostic applications.

In EUV lithography, imaging the EUV beam profiles is essential to ensuring consistent quality and precision in semiconductor chip manufacturing. Diamond single crystals are not suitable for this application due to their limited size (typically 3 × 3 mm^2^) and considerable thickness (typically 0.5 mm). To overcome these limitations, thin uniform FND films prepared by electrospray deposition have been developed as substitutes.^2^ The method exploits a high electric field to generate a fine mist of charged droplets containing FNDs, which are then deposited onto substrates.^50^ As charged droplets naturally disperse in the air, the FND thickness and deposition area can be precisely controlled by adjusting the spray time and plume size. A homogeneous film of 1 μm thick and 10 × 10 mm^2^ in area can be uniformly fabricated on an electrically conductive substrate, such as a glass slide coated with indium tin oxide (ITO), as shown in Figure 4a. The film thickness is approximately 1 μm, with a uniformity of ±100 nm across the sample (Figure 4b). This thickness is adequate for the detection of EUV radiation over the wavelength range of 1–120 nm, given that its penetration depth in diamond is less than 1 μm.

**Figure 4:**
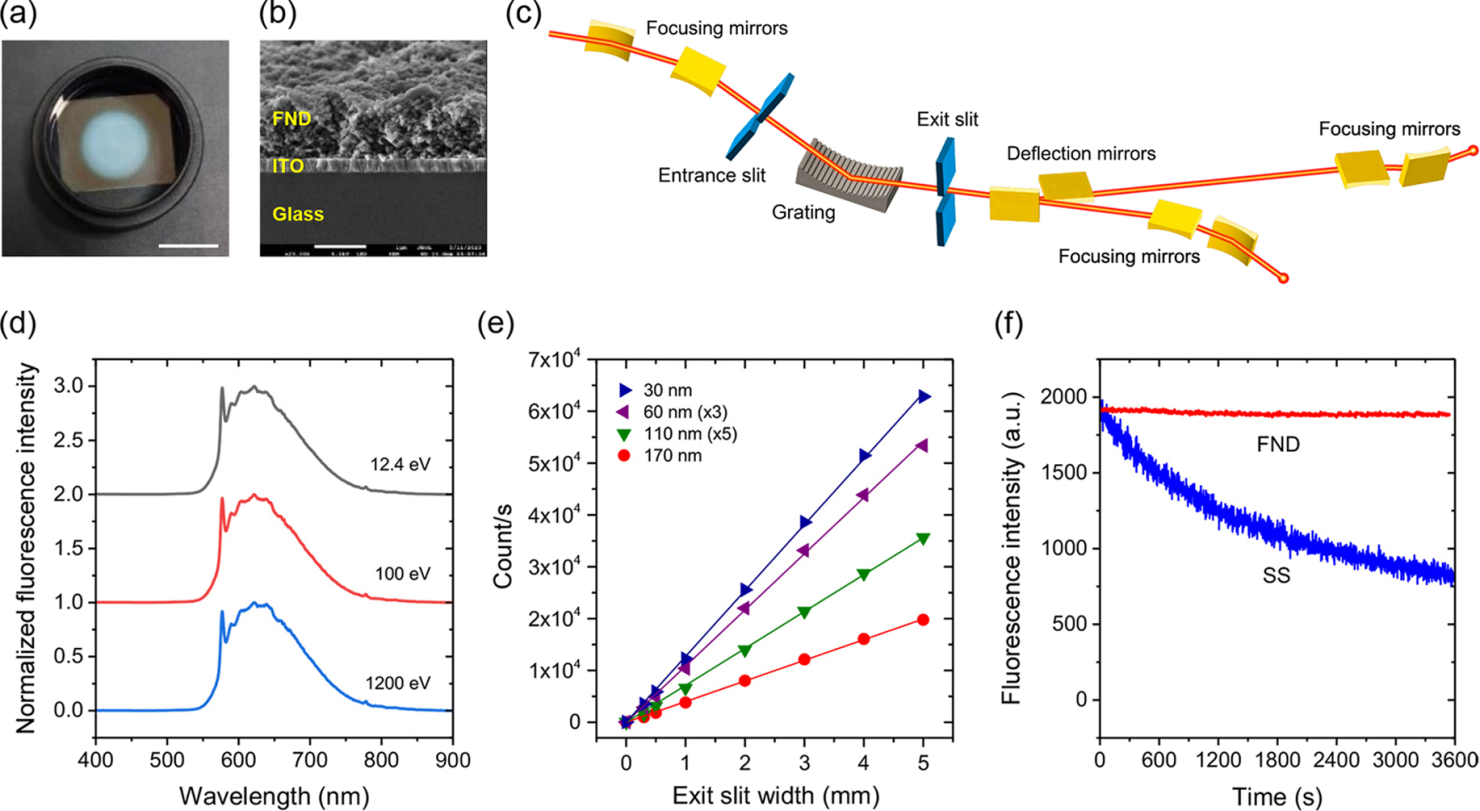
(a) Photograph and (b) cross-sectional scanning electron microscopy image of an FND film prepared by electrospray deposition and used as an EUV scintillator. Scale bars: 1 cm (a) and 1 μm (b). (c) Simplified optical layout of a synchrotron radiation beamline used for EUV sensing and imaging. Red dots at the right ends of the beams indicate the sample positions. (d) Fluorescence spectra of FNDs excited with synchrotron radiation of 12.4, 100, and 1200 eV in energy. (e) Dependence of the fluorescence intensities of FNDs on synchrotron radiation beam fluxes, controlled with the exit slit in the beamline. (f) Photostability tests of FND and sodium salicylate (SS) sensors exposed continuously to EUV radiation of 60 nm in wavelength for 1 h.

The EUV sensing capability of FNDs is demonstrated using synchrotron radiation over the energy range of 6.2–1200 eV or the wavelength range of 1–200 nm.^2,45–48^ The radiation is generated by deflecting electrons traveling near the speed of light with magnetic fields. A grating selects the wavelength of interest, while two slits control the photon flux (Figure 4c). Consistent with the mechanism illustrated in Figure 1d, exposure of FNDs to these radiations results in only NV^0^ fluorescence (Figure 4d). The response is highly linear (Figure 4e) and remains stable under continuous EUV illumination. The fluorescence intensity changes by less than 2% after 1 h of radiation exposure at a beam flux of appproximately 1 x 10^11^ photons/s (Figure 4f). In contrast, the performance of sodium salicylate, a widely used scintillator for synchrotron radiation, decreases by over 40% under the same irradiation conditions.^46^ Furthermore, temporal response tests, performed by randomly opening and closing the beamline shuttle, show negligible afterglow (i.e., persistent luminescence). These results collectively demonstrate the superior performance of FND over sodium salicylate as an EUV scintillator. The new scintillators have been successfully employed to record the absorption spectra of gaseous O_2_ over the wavelength range of 110–200 nm.^46^

By using FNDs, the EUV sensing device can be tailored to meet specific requirements or adapt to specialized detection applications. One novel application of the FND-coated plate is as an EUV viewing card for synchrotron radiation beams.^47^ Alternatively, the device can be coupled with a lens system and then a visible camera to precisely monitor the intensity profiles of an EUV beam and capture its fluorescence images in real time. Additionally, this diamond-based EUV imaging system (DEUVIS in short) can be miniaturized by coating FNDs onto a fiber-optic plate (FOP) mounted directly on the optical camera’s sensor. Figure 5a shows an optical image of the FOP used in this fiber-coupled device. It has a numerical aperture of 1 and a resolving power of 6 μm. Fibers in the plate are fused to form a vacuum-tight glass plate, which can be readily coated with an ITO layer and subsequently with FNDs by electrospray deposition (Figure 5b). For EUV radiation at a wavelength of 13.5 nm (91.8 eV energy), the fiber-coupled DEUVIS (Figure 5c) exhibits a noise-equivalent power density of 0.25 μW/cm^2^·Hz^1/2^, about 8× lower than that of the lens-coupled DEUVIS.^47^ This enhanced sensitivity makes the fiber-coupled device particularly useful for beam monitoring and profiling of EUV and even soft X-ray radiations from various sources, including laser-produced plasma, and high-order harmonic generation.^51–53^ The device has been successfully employed to investigate the beam profiles of synchrotron radiation over the photon energy range of 300–1100 eV (Figure 5d). The ability to image soft X-ray beams underscores the promise of FND coatings as a cost-effective, versatile diagnostic tool for advancing next-generation nanoelectronics.

**Figure 5:**
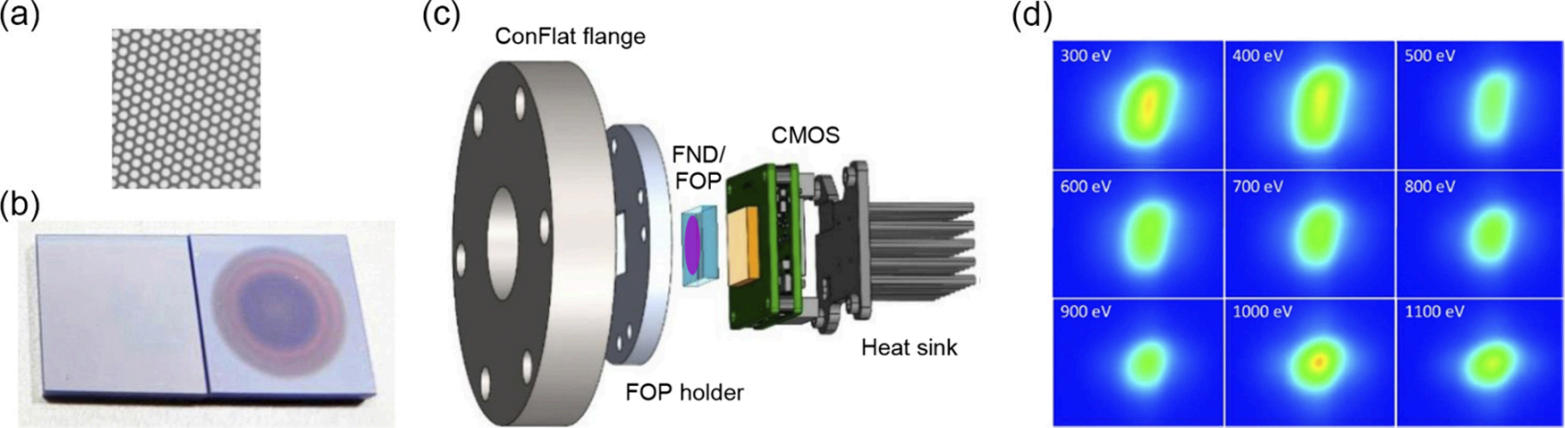
(a) Bright-field image of an FOP without FND coating. The individual fibers fused together in the FOP have a diameter of 6 μm. (b) Photograph of two FOPs without (left) and with (right) FND coatings prepared by electrospray deposition. FOP dimensions: 14 × 14 mm^2^. (c) Exploded view of a fiber-coupled DEUVIS equipped with a complementary metal oxide semiconductor (CMOS) camera. (d) Beam profiles of synchrotron radiation with photon energy varying over 300–1100 eV, imaged using the fiber-coupled DEUVIS. Image dimensions: 4.97 × 3.73 mm.

## 5 Semiconductor Device Analysis

Semiconductor-enabled nanoelectronics is a groundbreaking technology that transforms modern society. It allows for the creation of faster, smaller, and more energy-efficient devices, supporting the ongoing miniaturization of electronic components.^54^ However, as device sizes decrease to the nanometer scale, even the tiniest defects can impair the performance or cause complete system failure. Therefore, the development and implementation of robust, real-time diagnostic techniques for these devices are crucial. Pioneering studies in this field include the use of NV magnetometers with bulk diamonds for electric current imaging to detect failures in integrated circuits.^55,56^ Recent technological advances using NV^–^ centers in FNDs for temperature and magnetic sensing further offer promising solutions to these challenges.^57,58^

Thermometry is a fundamental tool in semiconductor device analysis, and its importance continues to grow as electronic components shrink in size and become more densely packed, posing greater challenges for thermal management.^59^ To address this issue, an FND-based lock-in photoluminescence (PL) thermography technique has been developed to enable wide-field, real-time nanoscale thermometry of operando semiconductor devices, such as bipolar functional transistors and field-effect transistors (FETs).^3^ In this approach, an ultrathin FND film (near-monolayer or ∼100 nm thick) is deposited onto targeted semiconductor devices via electrospray deposition, forming a dense array of nanoscale temperature sensors. The method overcomes the common limitations of drop-casting, which often leads to an uneven distribution of FNDs on the device surface due to coffee-ring effects and particle aggregation during solvent evaporation,^60^ thereby compromising image quality and obscuring structural details.

The FND-based lock-in PL thermography operates on the principle that the PL intensity of NV centers decreases with increasing temperature due to an enhancement in the nonradiative decay rate during energy relaxation (e.g., Figure 1c for NV^–^). As a result, the PL quantum yields are reduced and the fluorescence lifetimes are shortened.^61^ However, to accurately measure PL intensity changes in an ultrathin FND film with nanoscale resolution, the positioning of the sample must be adequately maintained over time to ensure consistent measurements. Figure 6a shows a schematic diagram of an inverted fluorescence microscope system equipped with automatic focus correction to achieve this goal. Using this setup, temperature-dependent PL intensity measurements for a near-monolayer of FNDs on a Si wafer reveal a variation of −0.090%/K over the 300–400 K range (Figures 6b and 6c). Although the thermal effect is small, it is readily measurable with lock-in techniques. Both NV^0^ and NV^–^ centers contribute to the observed fluorescence when the films are excited with green light at 550 nm.

**Figure 6:**
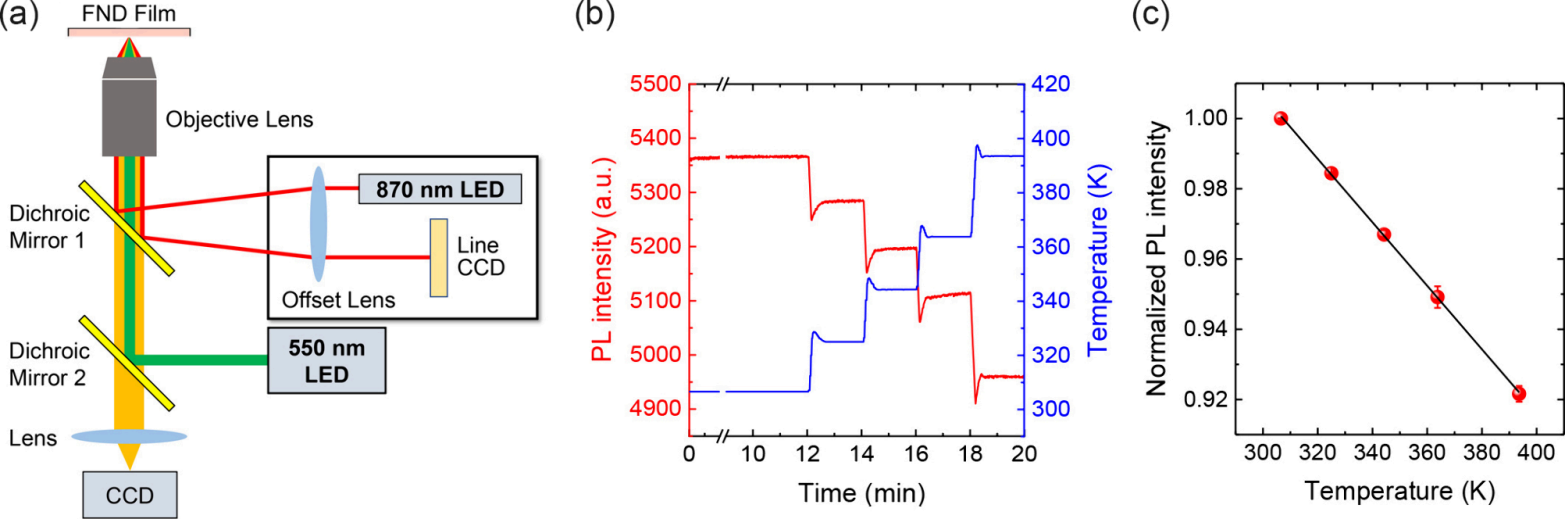
(a) Schematic diagram of the experimental setup for FND-based lock-in PL thermography. The setup consists of an inverted fluorescence microscope equipped with an autofocusing system to correct for sample drift. LED: light-emitting diode, CCD: charge-coupled device. (b) Time traces of substrate temperature and PL intensity upon heating of near-monolayer FNDs coated on a Si wafer. (c) PL intensity versus substrate temperature for near-monolayer FNDs coated on a Si wafer. Reproduced with permission from ref 3. Copyright 2026 Wiley.

The FND-based lock-in PL thermography has been employed to identify point defects and detect localized heating in operando semiconductor devices. This is accomplished by switching the devices on and off using current pulses. Fast Fourier transform analysis is then applied to the time-varying PL signals at each pixel to generate temperature change (Δ*T*) images. In this proof-of-principle experiment, the semiconductor device consists of a P-channel vertical double-diffused FET (Figure 7a), in which the gate voltage controls the current flowing from the source to the drain. A gate voltage of −60 V is first applied to the transistor’s gate terminal to create defects in the device.^62^ Lock-in IR thermography clearly identifies a distinct hot spot near the device boundary when the defective FET operates at a gate voltage of −20 V (Figure 7b). FND-based lock-in PL thermography also detects the same defect, showing a temperature increase about 10× higher than in its surrounding areas. The full width at half-maximum (fwhm) of the hot-spot thermal profile is 30.3 ± 0.3 μm (Figure 7c), with a peak localization precision of ±33 nm. By overlaying the corresponding bright-field and PL intensity images, the exact positions of the defects can be mapped in two dimensions. In comparison, the IR image reveals a Lorentzian-like peak with an fwhm of 67 ± 2 μm and a peak localization precision of ±0.7 μm, limited by its spatial resolution (Figure 7d). The exact temperature rise cannot be determined in IR thermometry due to the lack of emissivity information for the hot spot.

**Figure 7:**
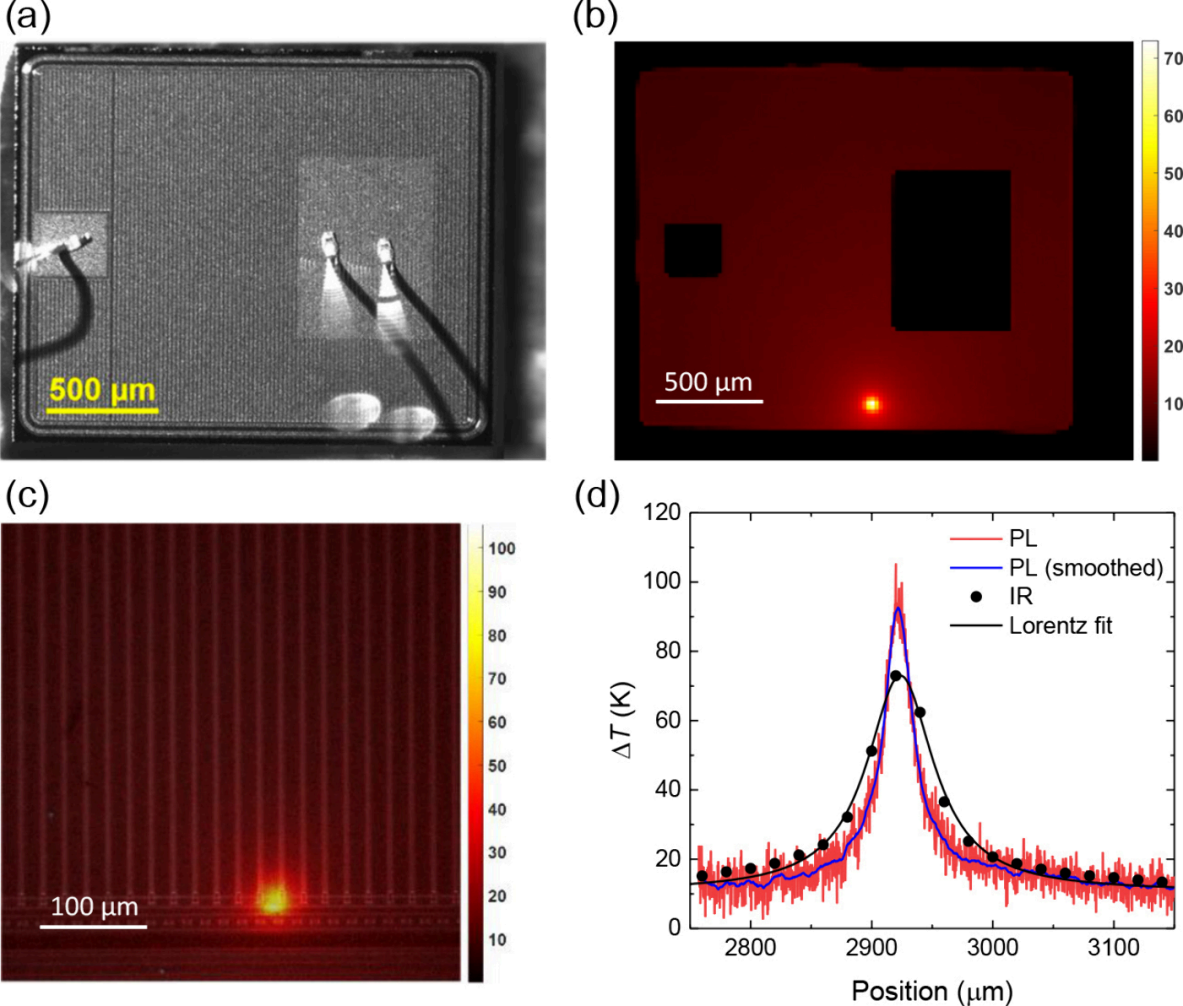
(a) Bright-field microscopy image of an FET enclosed in a metal can. (b) Lock-in IR thermography and (c) lock-in PL thermography of a defective FET coated with near-monolayer FNDs. Scale bar units: °C. (d) Comparison of hot-spot temperature line profiles measured by lock-in IR tomography and FND-based lock-in PL thermography. The blue curve represents data after 100-point smoothing. An emissivity of 0.46 is used in the IR thermographic measurements. Reproduced with permission from ref 3. Copyright 2026 Wiley.

In addition to the identification of point defects in PL images, this FND-based intensiometric method also enables real-time measurement of heat transfer dynamics.^3^ By continuously monitoring the temperature during the operation of an FND-coated bipolar functional transistor for 10 min, an effective junction-to-ambient thermal resistance (*R*_θJA_) of 99 ± 1 °C/W has been measured. The value matches the manufacturer’s specifications of *R*_θJA_ ≤ 292 °C/W, demonstrating the method’s accuracy and utility in quantifying key thermal parameters beyond imaging in semiconductor devices. While the current temporal resolution of 0.07 s is limited by the camera’s frame rate, it can be significantly improved with advanced imaging systems, such as lock-in cameras with response times as fast as 20 μs.^63^

## 6 Conclusions and Future Perspectives

FNDs are biocompatible quantum sensors with exceptional background-free detection capabilities. Integrating these sensors into immunoassays can enhance sensitivity, speed up results, and enable multiplexing in healthcare diagnostics; however, several challenges must be addressed to unlock their full potential. First, improvements in surface modification techniques for FNDs are essential to increase biomolecule attachment, reduce nonspecific binding, and improve assay reliability. Although surface modification of FNDs with polyethylene glycol is a common practice,^30^ encapsulating FNDs within lipid layers presents a promising alternative that merits further investigation.^64^ Second, a thorough evaluation of reproducibility, sensitivity, and specificity is essential before these assays can be widely adopted in clinical and commercial settings. Finally, as with any emerging diagnostic technology, standardization and regulatory approval will be critical hurdles for the broader implementation of FND-based immunoassays in both lateral-flow and microfiltration formats.

For beam diagnostics in photolithography, FNDs with NV centers offer several distinct advantages over conventional scintillators. First, they are chemically inert, nonhygroscopic, photostable, fast-responding, and produce high light output without afterglow. Additionally, low-cost, large-area EUV sensing and imaging devices can be facilely fabricated using FNDs as carbon-based scintillators, enabling real-time, in situ, and long-term beam diagnostics of EUV and soft X-ray radiations. Furthermore, FNDs that emit green fluorescence from N–V–N (or H3) centers are also available for the same application.^65^ However, before they are deployed in EUV lithography and at synchrotron radiation facilities, the chemical stability and physical durability of these sprayed FND films need further improvement. A simple and effective approach is to covalently cross-link FND particles within the films through post-treatment with oxygen plasma, which also helps to remove organic contaminants and regenerate clean film surfaces for repeated use. Lastly, comprehensive quality control and reproducibility tests are necessary to make the devices practically useful.

In semiconductor device analysis, IR thermography, thermoreflectance imaging, and Raman thermography are commonly employed techniques for thermal management and performance improvements.^66^ However, each method has its own advantages and limitations in resolution, sensitivity, and ease of use. To address these limitations, FND-based lock-in PL thermography has been developed as a versatile and user-friendly tool. Like IR thermography, it provides wide-field, real-time images with spatial resolution exceeding that of IR techniques by nearly 2 orders of magnitude, although its temperature measurement sensitivity is 10× lower. Enhancing the detection sensitivity remains an important goal for future work and can be addressed by increasing imaging frame rates to enable greater signal averaging.^63^ Combining this approach with FND-based magnetometry and thermometry using optically detected magnetic resonance represents a novel extension of this research.^60^ The integration of these platforms constitutes a significant advance in expanding the functional scope of FNDs as nanoscale quantum sensors for next-generation semiconductor technologies.
